# The application of lateral-rectus approach on toddlers’ unstable pelvic fractures

**DOI:** 10.1186/s12891-020-3172-1

**Published:** 2020-03-04

**Authors:** Yuancheng Liu, Xiaorui Zhan, Fuming Huang, Xiangyuan Wen, Yuhui Chen, Cheng Yang, Shicai Fan

**Affiliations:** The Third Affiliated Hospital of Southern Medical University, No.183 Zhongshan Avenue West, Tianhe District, Guangzhou, 510630 China

**Keywords:** Pelvic fracture, Toddlers, Lateral-rectus approach

## Abstract

**Background:**

Pelvic fractures are rare in toddlers but are often associated with other injuries that make treatment difficult. Conservative treatment has been used with moderate success, but it is unclear if surgical correction could confer additional benefits and improve patient outcomes. The purpose of this study was to report authors’ experience using the lateral-rectus approach (LRA) for surgical correction of unstable pelvic fractures in two toddlers.

**Methods:**

We retrospectively analyzed the cases of two toddlers with unstable pelvic fractures who underwent surgery through the LRA between April 2016 and October 2018. Patients’ characteristics, fracture type, mechanism of injury, Injury Severity Score (ISS), operative time, intra-operative blood loss, and post-operative complications were assessed. Pelvic asymmetry, degree of deformity, Cole scoring criteria and modified Barthel Index (MBI) were used to evaluate radiographic and functional outcomes.

**Results:**

Successful surgical treatment was performed using the LRA, external fixation, and sacroiliac screw fixation. Surgery duration was 180 min on average, with an average intra-operative bleeding of 250 ml. There were no iatrogenic nerve injuries or infections. Pelvic asymmetry a week after surgery was 0.5 cm on average and dropped to 0.3 cm on average at the end of the follow-up period. The deformity index of the pelvis dropped from an average of 0.035 a week after surgery to 0.02 at the end of the follow-up period. The mean MBI was 100 in the last follow-up, and Cole scoring criteria categorized both patients as being in excellent condition. All patients achieved radiological bone union without discrepancy in length of the lower limbs. Neither patient had loss of reduction nor evidence of low back pain during the mean follow-up period of 22 months.

**Conclusions:**

Pelvic fracture in toddlers is rare, and surgical treatment requires careful consideration. The lateral-rectus approach was proven as a viable alternative for managing unstable pelvic fractures in toddlers, with minimal blood loss and risk of nerve injury. Furthermore, anterior external fixation and posterior sacroiliac screw fixation would be adequate for this population, with excellent final outcome.

## Background

Pelvic fracture in children is rare, with a reported incidence of 0.2–2% of all fractures in children [1, 2]. Up to 10% of pediatric pelvic fractures are unstable [3]. These fractures usually occur as a result of pedestrians being hit by cars and are often accompanied by injuries to the musculoskeletal system, genitourinary system, abdominal viscera, and central nervous system [4]. Although the incidence of pelvic fractures in children is low, associated mortality rates are reportedly as high as 25% [5–7]. The cause of death in children with pelvic fractures is mainly due to combined injuries, and not the fracture itself [8].

However, controversy still exists regarding the treatment of pelvic fractures in children. The traditional view is that conservative treatment involving bed rest, traction, and casting can achieve good results because of the remodeling potential in children [9]. Recent studies have shown that the remodeling ability of children after pelvic fracture is suboptimal [10, 11]. Complications after conservative treatment, such as pelvic asymmetry, non-structural scoliosis, lower back pain, discrepancy in length of the lower limbs, and claudication when walking seriously affect the quality of life of the children [4, 10]. Therefore, it is becoming increasingly accepted that surgically repairing unstable or displaced pelvic fractures, regardless of patient age or skeletal maturity, is beneficial [12].

This study aimed to report the authors’ experience of using the lateral-rectus approach (LRA) for surgical correction of unstable pelvic fractures in two toddlers.

## Methods

After receiving approval from the Ethics Committee, we evaluated the cases of two female toddlers who underwent surgical repair for unstable pelvic fractures by the same trauma orthopedic specialist between April 2016 and October 2018. Both patients were hit by cars and received primary care at the emergency ward of a local institution immediately following the trauma. One was referred to our department for final treatment because of the complexity of her injuries. Due to the difficulty of transfer, another patient was operated on by the same trauma orthopedic specialist after her condition was stabilized.

### Perioperative management

Upon admission of both patients, the multidisciplinary physicians comprehensively evaluated their conditions, processed it according to the Advanced Trauma Life Support (ATLS) principle, and treated any associated injuries. Both patients underwent computed tomography (CT) scanning of the chest, abdomen, and pelvis.

**Fig. 1:**
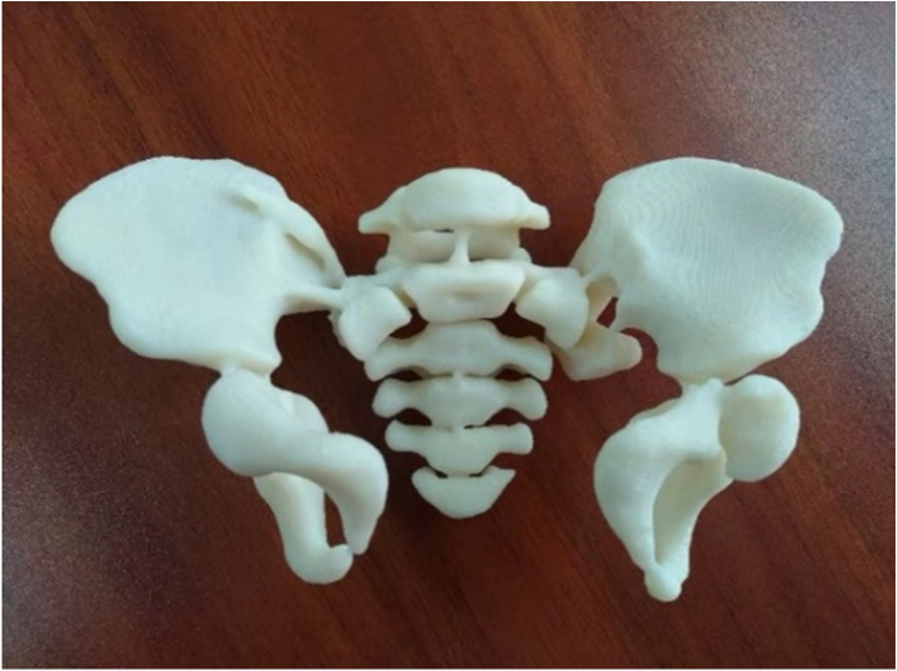
1:1 3D pelvic models

Pelvic CT data were saved in DICOM format and imported into Mimics software (Materialise, 21.0) for three-dimensional (3D) reconstruction. Preoperative planning was performed using 3D visualization technology. Firstly, the main part of the pelvic ring, with the axial bone as the reference, undisplaced segment, and the displaced fractures divided into different 3D objects. The displaced fractures were rotated and translated several times, and the pelvic ring was simulated and reduced on the virtual model. Cylinders were used to simulate the insertion, position, depth, and direction of the screws. Finally, 1:1 3D pelvic models were manufactured by 3D printing (Stratasys Dimension 1200es) to verify the feasibility of the surgical plan (Fig. 1).

A broad-spectrum antibiotic was given 30 min before the operation. One of the patients had a longer surgery that exceeded 180 min; therefore, the antibiotic was re-administered to this patient. An indwelling drainage tube was used after the operation and removed the day after the surgery. Liquid diet was allowed after anal exsufflation. Patients were encouraged to perform functional exercises of both lower limbs without weight bearing 7 days after surgery. Patients were permitted to resume partial-weight bearing exercises at 4 to 6 weeks and full-weight bearing at 8 to 12 weeks postoperatively.

### Operations

**Fig. 2:**
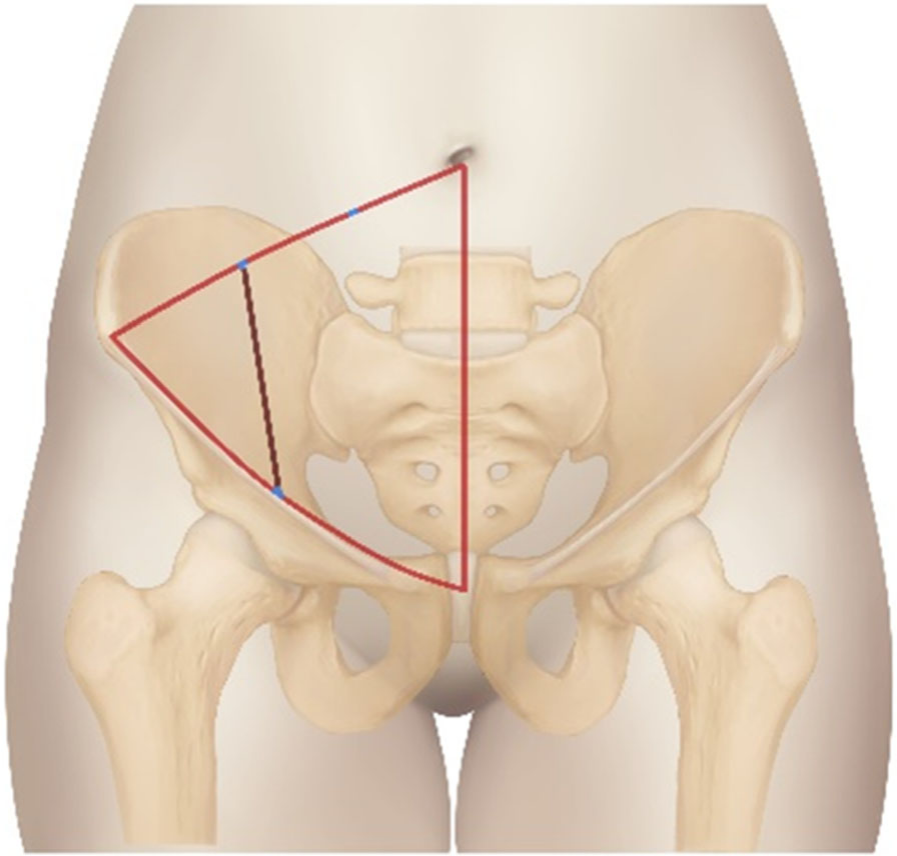
The incision of the lateral-rectus approach

**Fig. 3:**
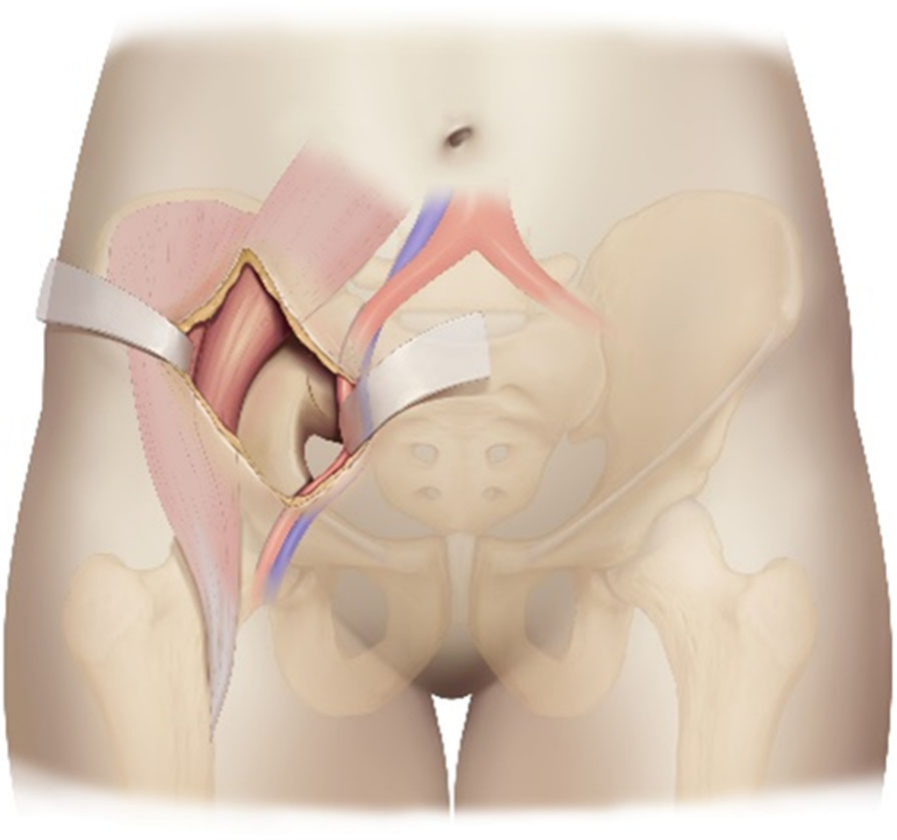
The exposure of the sacroiliac joint

After general anesthesia with tracheal intubation, patients were placed in the supine position on a radiolucent operating table. The patient’s lower limbs, perineum and abdomen were disinfected, and the lower limbs were bandaged for intraoperative traction. The LRA incision started from the point located two thirds of the distance between the umbilicus and anterior superior iliac spine, and ended at the midpoint of the inguinal ligament (Fig. 2). The surface projection of this approach corresponded to the sacroiliac joint. After splitting the obliquus externus abdominis, obliquus internus abdominis, and transverse abdominis, the peritoneum and internal organs were mobilized to completely expose the sacroiliac joint and sacral wing through the surgical window between the iliac vessels and iliopsoas muscle (Fig. 3). The soft tissue around the fracture site was removed. The fracture and dislocation of the posterior ring were reduced, combined with lower limb traction and prying reduction. A Kirschner wire was used for temporary fixation of the posterior ring. If C-arm fluoroscopy showed satisfactory reduction, the posterior pelvic ring was fixed with a sacroiliac screw. Meanwhile, the anterior ring was fixed externally, and a cannulated screw was added when necessary. After reduction and fixation, the operation cavity was cleansed, and hemostasis was stopped. The operation cavity was closed after placing a drainage tube.

### Follow-up and evaluation criteria

**Fig. 4:**
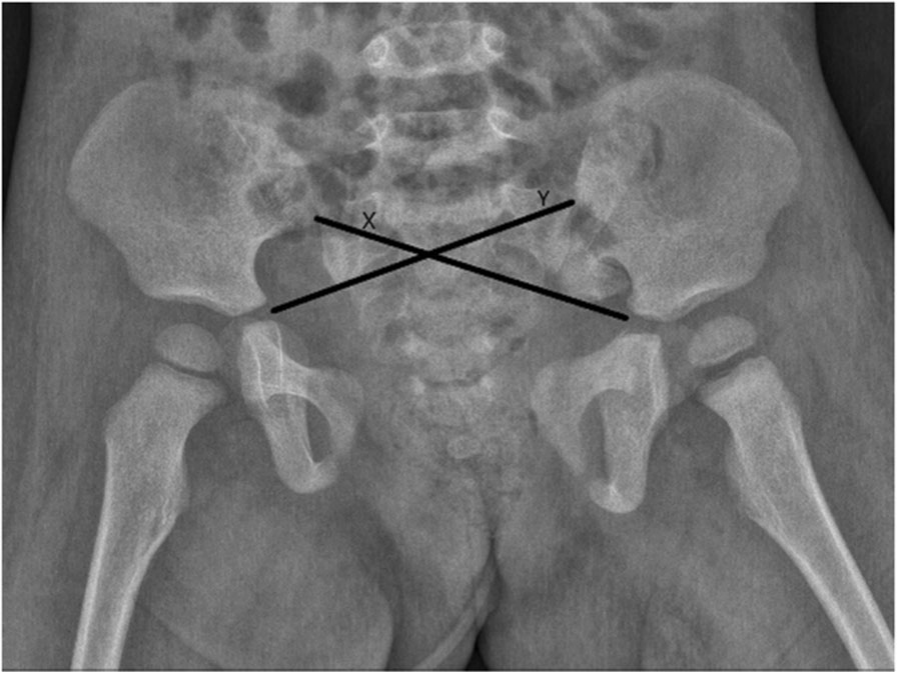
Keshishyan et al. method of measuring pelvic asymmetry. The difference of diagonals (X – Y cm) and the deformity index which corresponds to the difference in the diagonals divided by the sum of the diagonals show the pelvic asymmetry

Radiographic and functional assessments were performed during the follow-up appointments. The method proposed by Keshishyan [13] was used to evaluate the quality of reduction. The distance from the low border of the sacroiliac joint to the middle of the internal contour at the bottom of the contralateral acetabulum was measured on a standard pelvic anteroposterior X-ray radiograph. The difference of diagonals and the deformity index, which corresponds to the difference in the diagonals divided by the sum of the diagonals, indicated show pelvic asymmetry (Fig. 4). Functional outcome was evaluated using surveys including the Cole [14] scoring criteria and modified Barthel Index (MBI) [15]. All evaluations were assessed by two independent orthopedic surgeons who were not involved in the definitive care or surgery.

## Results

Patient clinical data are shown in Table 1. The Tile [16] classification was used to assess the cases, and produced the following distribution: C1.2(one case); C3(one case). Both fractures were classified as Type IV according to the Torode and Zieg [17] classification system. In addition, both patients had multiple associated injuries, including pulmonary contusion (*n* = 2), perineal laceration (*n* = 1), and inguinal laceration (*n* = 1). Both patients had ISS scores of 19 when at admission. Both were referred to ICU (Intensive Care Unit) for treatment due to the complexity of their conditions. Hemorrhagic shock occurred in both children, requiring blood transfusion, fluid resuscitation, and urgent pelvic stabilization by pelvic binder. However, angiography was not required. The patient with a perineal laceration was treated with surgical debridement and antibiotics. The patient with an inguinal laceration was treated with surgery and antibiotics, but progressed to a wound infection during hospitalization.

**Table 1:** Patient clinical data

Case	Sex	Age-ranges (years)	Tile classification	Torode and Zieg classification	Cause of the accident	Associated injuries	Injury Severe Score (ISS)
1	Girl	1~3	C3	IV	Pedestrian hit by a car	pulmonary contusion, inguinal laceration	19
2	Girl	1~3	C1.2	IV	Pedestrian hit by a car	pulmonary contusion, perineal laceration	19

After clinical stabilization, one patient was transferred to our hospital, and surgery was performed 13 days after the initial injury. The other patient was treated surgically by the same orthopedic specialist in the local hospital 25 days after the injury. Both patients underwent successful operation via the LRA. External fixations and sacroiliac screws were used for fixation, and a cannulated screw was added to fix pubic symphysis separation in one case. The average surgical duration was 180 min, and the average intra-operative bleeding was 250 ml. However, considering that the circulatory state and metabolic homeostasis of these patients are fragile during the perioperative period, 2 units of erythrocyte suspension were transfused slowly during surgery.

Preoperative radiographic assessment of pelvic asymmetry was not available in one case, and both toddlers received direct pelvic CT examinations. The results of the postoperative and final follow-up pelvic asymmetry and deformity index of the pelvis are shown in Table 2.

**Table 2:** Results of the treatment administered

Case	Time from injury to surgery (days)	Operative time (minutes)	Intra-operative blood loss (ml)	Follow-up period (month)	Post-op pelvic asymmetry (cm)	Post-op Deformity index	Pelvic asymmetry in last follow-up (cm)	Deformity index in last follow-up	modified Barthel Index (MBI)	Cole score
1	13	240	300	10	0.4	0.03	0.3	0.02	100	excellent
2	25	120	200	34	0.6	0.04	0.3	0.02	100	excellent

Both patients showed excellent clinical outcomes. No screw loosening, reduction loss, infection of the incision, or deep vein thrombosis occurred after the operation. No injury to the lumbosacral plexus or iliac vessels occurred in either case. The average MBI evaluated at the last follow-up was 100. The Cole scores evaluated at the end of follow-up were excellent in both cases. All external fixators were removed within 6 weeks after surgery, and all internal fixators were removed approximately 3 months after surgery. During a mean follow-up of 22 months, all the patients had gained bone union, and there was no evidence of pain on palpation of the pelvis, abnormal gait, discrepancy in leg length, or scoliosis during the physical examination. Results of the treatment administered are shown in Table 2.

**Fig. 5:**
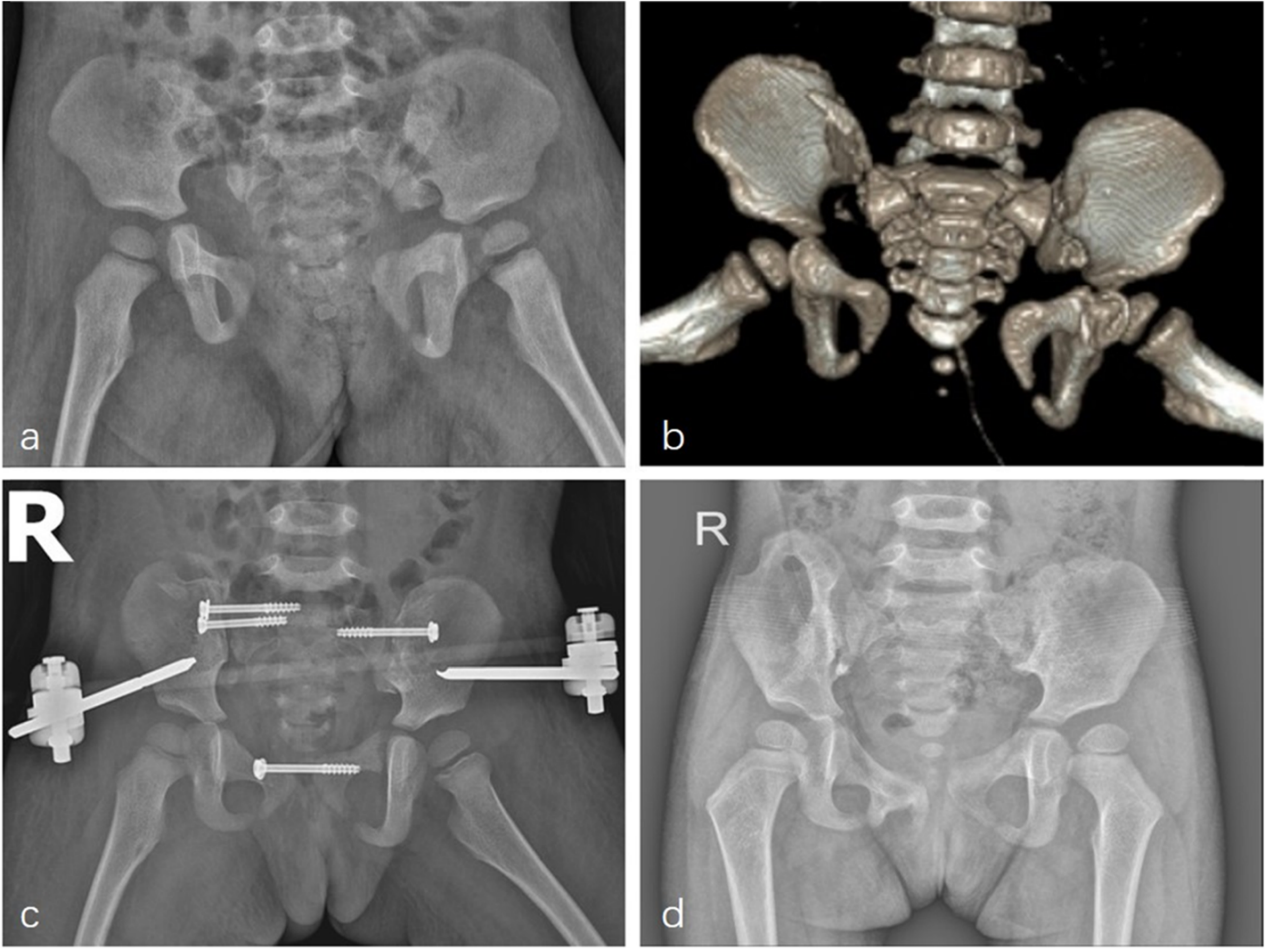
Case 1, **a**. Preoperative X-ray; **b**. Preoperative CT with 3 D reconstruction; **c**. postoperative X-ray; **d**. X-ray in the final follow-up

**Fig. 6:**
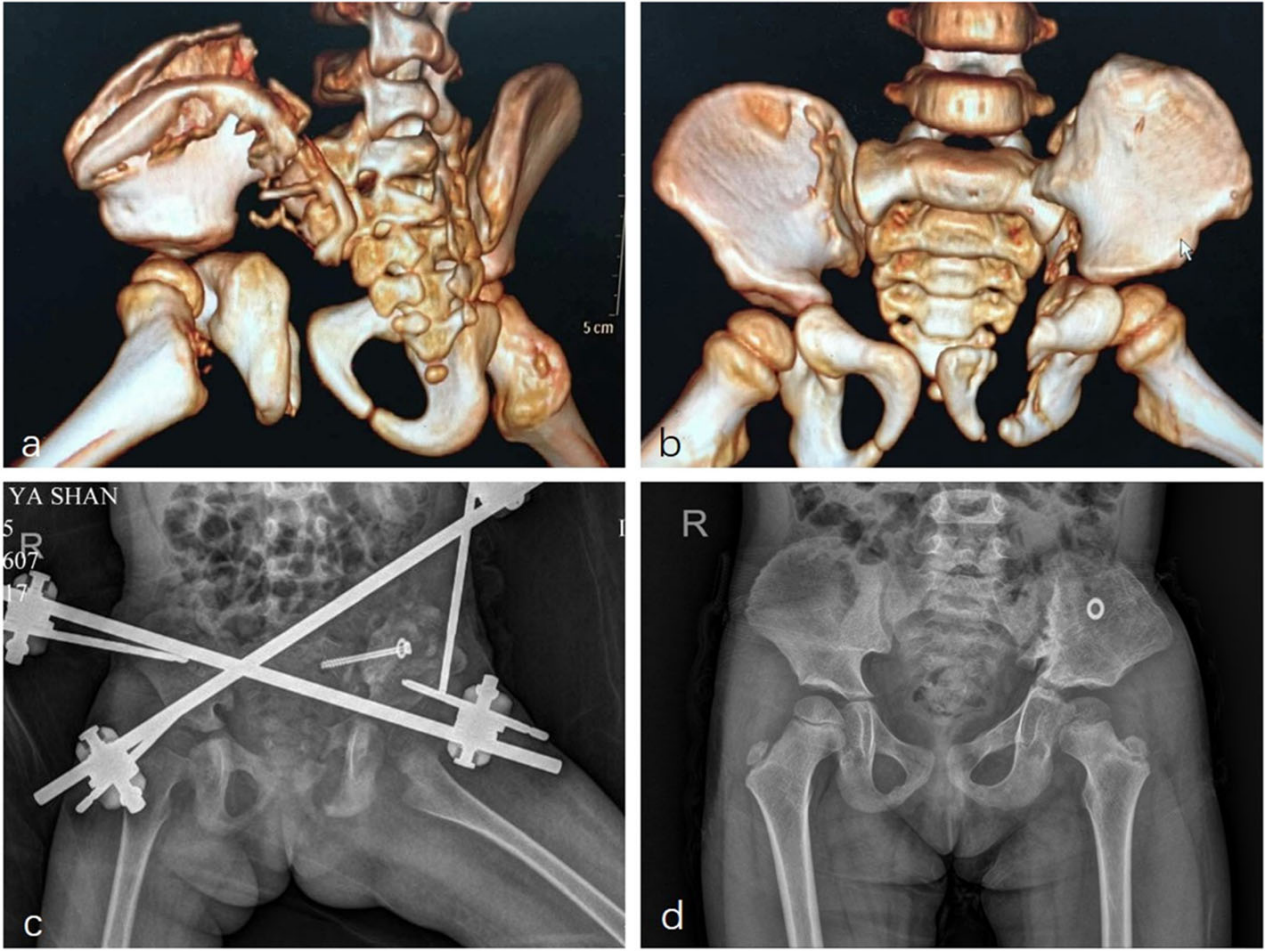
Case 2, **a**~**b**. Preoperative CT with 3 D reconstruction; **c**. postoperative X-ray; **d**. X-ray in the final follow-up

Figures 5 and 6 show X-rays and CT scans from both patients.

## Discussion

There is more cartilage and a thicker periosteum in the pediatric pelvis [12, 18]. Meanwhile, pediatric sacroiliac and pubic symphyseal joints are wider and thicker than adults, allowing them to absorb greater perturbations without disruption [12]. Unstable pelvic fractures are relatively rare, especially in pediatric patients, and a higher energy mechanism is required for pelvic fracture to occur in children. However, pelvic fractures frequently occur with other life-threatening injuries, which should be taken into account when planning treatment [3, 19]. Grisoni [20] observed that pediatric pelvic fractures were associated with less blood loss compared to adult pediatric pelvic fractures because the thicker periosteum can contain and tamponade bleeding. Additionally, pediatric vasculature, which is more vasoreactive than adult vasculature, can effectively constrict the bleeding. However, both cases of this study experienced hemorrhagic shock after injury, requiring blood transfusion and fluid resuscitation. This suggests that it is necessary to use CT examination to exclude the liver, spleen, and other potentially life-threatening injuries when an unstable pelvic fracture occurs, despite evidence that radiation exposure caused by CT examination may have adverse effects in children [12].

Silber and Flynn [21] divided pelvic fractures in children into mature and immature groups according to the state of the triradiate cartilage. The triradiate cartilage closes at 14 years of age in boys and at 12 years of age in girls, on average. For children and adolescents with pelvic fractures in which the triradiate cartilage has closed, the treatment principle is similar to that of adults.

According to the traditional view, pelvic fractures in children with open triradiate cartilage often do not require surgical treatment because of their good remodeling ability [9]. However, increasing evidence shows that non-surgical treatment can lead to poor prognosis in children with unstable fractures [22]. One possible reason is that due to the anatomical characteristics of children’s pelvis, sacroiliac joints have higher risk of anterior dislocation after injury, often accompanied with pubic symphysis separation [23]. Another possible reason is that the pediatric pelvic fractures are often caused after experiencing severe trauma, which also causes severe bone and cartilage damage. Surgical reduction and fixation is likely necessary for unstable pelvic fractures in children with open triradiate cartilage [12]. There are few pertinent studies regarding surgical treatment of pelvic fractures in children due to low incidence and the influence of traditional views. Most studies are case reports and retrospective studies with small sample sizes, and there is a lack of high-quality evidence to guide clinicians when making decisions regarding surgical intervention.

Rotationally unstable fractures (Tile B) are the most common type of pelvic fracture in children due to the mechanism of injury. External fixation and six-hole reconstruction plates were suggested to fix the anterior ring of the pelvis [3]. For vertically and rotationally unstable fractures (Tile C), Smith [24] showed that patients treated with combined anterior and posterior fixation had better outcomes than those treated with anterior external fixation alone. It has been reported that the posterior pelvic ring can be fixed with plates and screws [25]. However, the removal of secondary internal fixation is relatively traumatic, indicating that sacroiliac screws may be a better choice [19]. Sacroiliac screws can be inserted percutaneously when posterior ring injuries can be adequately reduced through closed methods. In this study, the posterior ring was fixed with sacroiliac screws, while the anterior ring was fixed with externally, and a cannulated screw was added to enhance stability of anterior ring in one patient.

Due to the occurrence of other injuries with pelvic fractures, it is often difficult for the initial hospitals to treat the pelvic fractures at early stages. When the condition of the child becomes stable, they pelvic fractures are already considered to be late stage. At present, the anterior ilioinguinal approach is frequently used to expose and reduce posterior pelvic ring injuries in children [3]. However, the ilioinguinal approach has several disadvantages: [1] The surgical trauma of the approach is severe [2]; It may damage the epiphysis of the ilium wing, leading to pelvic dysplasia; In addition, compared with the LRA, the ilioinguinal approach is more lateral, in which exposing the sacroiliac joint and performing reduction and fixation is relatively difficult from the side compared to the approach from the front [3]. As a viable alternative to the ilioinguinal approach, LRA has been used in pelvic acetabular fracture repair with good results [26].

This is the first study, to our knowledge, to apply LRA to pelvic fractures in children. In case 1 of Tile C pelvic fracture was corrected with bilateral LRA. The sacroiliac joint can also be exposed clearly through this approach, permitting effective reduction of the posterior ring. Sacroiliac screws can also be placed under direct vision to avoid vascular and nerve injury.

Most studies have adopted Keshishyan’s method to evaluate the radiographic outcomes of pelvic fractures in children [4, 27]. However, for the Tile C fracture in case 1, both the bilateral sacroiliac joints were damaged, this method could not reflect the degree of injury well, and it cannot accurately evaluate the quality of surgical reduction. Therefore, in addition to preoperative pelvic CT examination to fully assess the injury, postoperative pelvic CT examination to evaluate the efficacy of the surgery may be necessary.

## Conclusion

Pelvic fractures in toddlers are rare, and surgical treatment requires careful consideration. The lateral-rectus approach was proven as a good option for managing unstable pelvic fractures in toddlers, with sufficient exposure but minimal blood loss and risk of nerve injury. We believe that anterior external fixation and posterior sacroiliac screw fixation would be adequate for treating this type of injury in toddlers, with excellent final outcome.

## Data Availability

The datasets generated and/or analyzed during the current study are available from the corresponding author by reasonable request.

## References

[1] Chotai N, Alazzawi S, Zehra SS, Barry M (2018). Paediatric pelvic fractures: a review of 2 cohorts over 22 years. Injury.

[2] Holden CP, Holman J, Herman MJ (2007). Pediatric pelvic fractures. J Am Acad Orthop Surg.

[3] Wharton RMH, Trowbridge S, Simpson A, Sarraf KM, Jabbar Y (2019). Anatomic, diagnostic and management challenges in paediatric pelvic injuries: a review. J Pediatr Orthop B.

[4] Guimaraes JA, Mendes PH, Vallim FC, Rocha LR, Rocha TH (2014). Surgical treatment for unstable pelvic fractures in skeletally immature patients. Injury.

[5] Leonard M, Ibrahim M, McKenna P, Boran S, McCormack D (2011). Paediatric pelvic ring fractures and associated injuries. Injury.

[6] Ismail N, Bellemare JF, Mollitt DL, DiScala C, Koeppel B (1996). Death from pelvic fracture: children are different. J Pediatr Surg.

[7] Banerjee S, Barry MJ, Paterson JM (2009). Paediatric pelvic fractures: 10 years experience in a trauma Centre. Injury.

[8] DeFrancesco CJ, Sankar WN (2017). Traumatic pelvic fractures in children and adolescents. Semin Pediatr Surg.

[9] Musemeche CA, Fischer RP, Cotler HB, Andrassy RJ (1987). Selective management of pediatric pelvic fractures: a conservative approach. J Pediatr Surg.

[10] Oransky M, Arduini M, Tortora M, Zoppi AR (2010). Surgical treatment of unstable pelvic fracture in children: long term results. Injury.

[11] Pascarella R, Bettuzzi C, Digennaro V (2013). Surgical treatment for pelvic ring fractures in pediatric and adolescence age. Musculoskelet Surg.

[12] Amorosa LF, Kloen P, Helfet DL (2014). High-energy pediatric pelvic and acetabular fractures. Orthop Clin North Am.

[13] RA K (1995). Pelvic polyfractures in children. Radiographic diagnosis and treatment. Clin Orthop Relat Res.

[14] JD C Outcome after fixation of unstable posterior pelvic ring injuries. Clin Orthop Relat Res 1996;329:160-179. 10.1097/00003086-199608000-00020 8769448

[15] S S (1989). Improving the sensitivity of the Barthel Index for stroke rehabilitation. J Clin Epidemiol.

[16] Pennal GF, Tile M, Waddell JP, Garside H. Pelvic disruption: assessment and classification. Clin Orthop Relat Res. 1980:12–21. 7418295

[17] Benjamin JS (2012). Pediatric pelvic fracture: a modification of a preexisting classification. J Pediatr Orthop.

[18] Oberc A, Sulko J (2018). Pelvic fracture of the sacroiliac joint region in a child - a case study and literature review. J Pediatr Orthop B.

[19] Scolaro JA, Firoozabadi R, Routt MLC (2018). Treatment of pediatric and adolescent pelvic ring injuries with percutaneous screw placement. J Pediatr Orthop.

[20] Grisoni N, Connor S, Marsh E, Thompson GH, Cooperman DR (2002). Pelvic fractures in a pediatric level I trauma center. J Orthop Trauma.

[21] Silber JS, Flynn JM (2002). Changing patterns of pediatric pelvic fractures with skeletal maturation: implications for classification and management. J Pediatr Orthop.

[22] Zwingmann J, Aghayev E, Sudkamp NP, Neumann M, Bode G (2015). Pelvic fractures in children results from the German pelvic trauma registry: a cohort study. Medicine.

[23] Zhang Q, Chen W, Liu H, Su Y, Pan J (2009). The anterior dislocation of the sacroiliac joint: a report of four cases and review of the literature and treatment algorism. Arch Orthop Trauma Surg.

[24] Smith W, Shurnas P, Morgan S, Agudelo J, Luszko G (2005). Clinical outcomes of unstable pelvic fractures in skeletally immature patients. The journal of bone and joint surgery. American volume.

[25] Shi Q, Wu WP, Han J, Dai SW, Tan W (2015). Fracture-dislocation of the sacroiliac joint with severely unstable fractures of the pelvis and femur in a 16-month-old patient: a case report. J Orthop Sci.

[26] Chen J, Liu H, Wang C, Lin X, Gu C (2019). Internal fixation of acetabular fractures in an older population using the lateral-rectus approach: short-term outcomes of a retrospective study. J Orthop Surg Res.

[27] Smith WR, Oakley M, Morgan SJ (2004). Pediatric pelvic fractures. J Pediatr Orthop.

